# A Comprehensive Mitochondrial Cytochrome c Oxidase Subunit II Gene (*cox2*)-Based Framework for Genotyping and Phylogenetic Analysis of *Pythium insidiosum* and Related Taxa

**DOI:** 10.3390/microorganisms14102299

**Published:** 2026-10-09

**Authors:** Nichapat Yurayart, Thidarat Rujirawat, Theerapong Krajaejun

**Affiliations:** 1 Department of Food Animal Clinical Sciences, Faculty of Veterinary Medicine, Khon Kaen University, Khon Kaen 40002, Thailand; 2 Offices of Health Science Research, Faculty of Medicine, Ramathibodi Hospital, Mahidol University, Bangkok 10400, Thailand; thidarat.ruj@mahidol.ac.th; 3 Department of Pathology, Faculty of Medicine, Ramathibodi Hospital, Mahidol University, Bangkok 10400, Thailand

**Keywords:** pythiosis, phylogenetics, genotyping, *cox2*, genetic diversity

## Abstract

*Pythium insidiosum* is an important oomycete pathogen that causes pythiosis, a severe infectious disease affecting humans and animals worldwide. Understanding its genetic diversity is essential for accurate species identification, genotyping, and molecular characterization. Although the ribosomal DNA internal transcribed spacer (ITS) region is widely used as a DNA barcode, its limited variability can restrict fine-scale genotyping, highlighting the need for alternative genetic markers. Previous studies demonstrated the utility of the mitochondrial cytochrome c oxidase subunit II gene (*cox2*) for molecular identification and phylogenetic analysis of *P. insidiosum*. However, a comprehensive global assessment of *cox2* sequence diversity has not been conducted. In this study, we established a comprehensive *cox2* sequence dataset representing diverse *P. insidiosum* isolates from multiple hosts and geographic regions. A total of 249 unique *cox2* sequences were analyzed, including 171 retrieved from the NCBI database and 78 newly generated from our culture collection. Using a standardized 387-bp *cox2* fragment, phylogenetic analysis resolved four major phylogenetic clades (I–IV), with additional subclade resolution. Sequence alignment identified an 18-nucleotide signature motif for genotype differentiation. A 98.5% sequence identity threshold was used as a practical guideline for sequence screening and species-level identification. Clade II was the most prevalent lineage, accounting for 120 of 249 isolates (48.2%), and was further resolved into two distinct subclades (IIa, *n* = 82; IIb, *n* = 38), indicating substantial genetic diversification within the clade. Collectively, these findings establish *cox2* as a robust molecular marker and provide a comprehensive reference dataset for *P. insidiosum* genotyping. This resource supports species identification, genotyping, and molecular characterization.

## 1. Introduction

*Pythium* is a diverse genus of oomycetes comprising numerous species adapted to a broad range of ecological niches, including soil, freshwater, agricultural, and aquatic environments [1]. Members of the genus exhibit diverse lifestyles, including free-living, plant-pathogenic, and animal- and human-pathogenic species [2]. The ecological versatility and genetic diversity observed across the genus underscore the importance of molecular approaches for species identification, phylogenetic characterization, and investigations of evolutionary relationships within *Pythium* and related taxa.

*Pythium insidiosum* de Cock et al. is an oomycete pathogen that causes pythiosis, a severe infectious disease affecting both humans and animals worldwide [3]. This organism thrives in freshwater environments and is particularly prevalent in tropical and subtropical regions where environmental conditions favor transmission [4,5]. Pythiosis is often difficult to diagnose and treat, resulting in severe clinical outcomes with high morbidity and mortality [6,7]. Understanding the genetic diversity of *P. insidiosum* is important for molecular characterization and accurate identification of this pathogen. Previous studies have identified multiple genotypes (clades I–IV), reflecting the evolutionary diversity of *P. insidiosum* [8,9], which supports more accurate molecular characterization and identification.

Molecular identification of *P. insidiosum* relies on DNA barcoding, which compares target sequences with reference databases [10]. The ribosomal DNA internal transcribed spacer region (ITS) has been widely used as a standard barcode for species-level identification, including in *P. insidiosum* [11]. However, the ITS region may lack sufficient variability for fine-scale genotyping and phylogenetic resolution in certain isolates [4], limiting its utility for detailed epidemiological studies. To address these limitations, alternative genetic markers have been explored. Among them, the mitochondrial cytochrome c oxidase genes (*cox1* and *cox2*) have shown promise for species identification and genotyping [4]. Robideau et al. demonstrated that both the mitochondrial cytochrome c oxidase subunit I gene (*cox1*) and the nuclear ITS region are effective and complementary DNA barcode markers for oomycete identification [12]. In *P. insidiosum*, ITS and mitochondrial cytochrome c oxidase subunit II gene (*cox2*) have been widely used for molecular identification, genotyping, and phylogenetic studies [4,13]. Previous investigations comparing ITS and *cox2* demonstrated that both markers recover similar major phylogenetic groupings, whereas *cox2* may provide higher phylogenetic resolution among closely related *P. insidiosum* strains [14]. Given its demonstrated utility, the availability of a relatively large number of publicly accessible sequences, and its suitable balance between conservation and variability, *cox2* represents a practical marker for large-scale comparative phylogenetic analyses and investigations of genetic diversity within *P. insidiosum*, while enabling reliable sequence alignment and sufficient phylogenetic informativeness for detecting and differentiating the pathogen [14].

Previous studies have demonstrated the utility of the *cox2* locus for molecular identification, genotyping, and phylogenetic analysis of *P. insidiosum* [4,8,14]. However, these studies were generally based on relatively limited collections of isolates or region-specific datasets and were primarily designed to establish the phylogenetic utility of the marker rather than to provide a comprehensive assessment of global *cox2* diversity. Consequently, available *cox2* data remain fragmented across individual studies and geographic regions, and a comprehensive global evaluation of *cox2* sequence diversity has not been conducted. In addition, the phylogenetic relationships among *P. insidiosum* lineages and closely related taxa have not been comprehensively evaluated using a large, integrated *cox2* sequence dataset representing diverse hosts, geographic regions, and phylogenetic lineages. To address these knowledge gaps, we compiled a comprehensive global dataset of *cox2* sequences from *P. insidiosum* and related species, including *Pythium destruens* Shipton and *Pythium periculosum* Vilela & L. Mend. Sequences were retrieved from the NCBI database and supplemented with newly generated sequences from our laboratory culture collection. The combined dataset was analyzed to characterize genetic diversity, phylogenetic relationships, and informative sequence variation within the *cox2* gene.

## 2. Materials and Methods

### 2.1. Retrieval of Pythium insidiosum Mitochondrial Cytochrome c Oxidase Subunit II Gene (cox2) Sequences from the NCBI Database

*Pythium insidiosum cox2* sequences were retrieved from the NCBI database on 22 March 2024 using BLAST searches (https://blast.ncbi.nlm.nih.gov/Blast.cgi) with four reference sequences representing the major genotyping clades: LC805695 (Clade I), LC553043 (Clade II), LC805721 (Clade III), and OM363736 (Clade IV). BLAST searches initially retrieved the top 1000 homologous sequences from the NCBI database. Candidate sequences were considered for further evaluation if they were annotated as *cox2* genes of *P. insidiosum*, *Pythium periculosum*, or *Pythium destruens*, or showed ≥98.5% sequence identity, a commonly accepted threshold for species identification [15]. Final dataset inclusion was determined after evaluation of sequence annotation, sequence quality, and phylogenetic relevance. Sequences annotated as *P. insidiosum* in the NCBI database were retained for phylogenetic analysis even when nucleotide identity was below 98.5%, as these sequences may represent genetically divergent lineages within the species and provide valuable information on intraspecific diversity and evolutionary relationships. When duplicate sequences from the same isolate were identified, only the highest-quality sequence, based on length and number of gaps, was retained for subsequent analyses.

### 2.2. Origins, DNA Extraction, and cox2 Sequencing of Additional P. insidiosum Isolates

To expand the dataset, *cox2* sequences were generated from 78 *P. insidiosum* isolates maintained in the Ramathibodi Hospital Culture Collection. These isolates were obtained from diverse hosts and geographic origins, as summarized in Supplementary Table S1. Information on the dates of deposition and duration of maintenance of individual isolates in the collection was not consistently available and was therefore not included. The isolates were subcultured on sterile cellophane membranes overlaid on Sabouraud dextrose agar (Sigma-Aldrich, St. Louis, MO, USA) for 7–10 days. Approximately 200 mg of actively growing biomass was harvested from each isolate for genomic DNA (gDNA) extraction using a salt extraction method [16]. PCR amplification of the *cox2* gene was performed in 50-µL reactions containing 100 ng gDNA, 0.4 µM of each oomycete-specific primer [FM58 (5′-CCACAAATTTCACTACATTGA-3′) and FM66 (5′-TAGGATTTCAAGATCCTGC-3′)], 0.2 mM dNTPs, 1.5 mM MgCl_2_, 1.25 U Taq DNA polymerase (Thermo Scientific, Waltham, MA, USA), and 1× KCl buffer, following established protocols [4]. Thermal cycling conditions consisted of initial denaturation at 94 °C for 5 min, followed by 35 cycles of denaturation at 94 °C for 60 s, annealing at 52 °C for 60 s, and extension at 72 °C for 60 s, with a final extension at 72 °C for 10 min.

Amplicons were analyzed using the QIAxcel Advanced system with capillary electrophoresis and a DNA screening kit (15–5000 bp range) and visualized using QIAxcel ScreenGel version 1.3.0 software (Qiagen, Hilden, Germany). PCR products were purified using a PCR purification kit (Qiagen, Germany) and sequenced with the same primer pair. All sequences were validated by BLAST search against the NCBI nucleotide database. The resulting *cox2* sequences were deposited in GenBank under accession numbers LC805689–LC805766.

### 2.3. Multiple Sequence Alignment and Phylogenetic Analysis

All *cox2* sequences from *P. insidiosum* and related taxa were aligned and trimmed using MUSCLE v3.8 and BMGE v1.12 within the NGPhylogeny.fr platform [17]. Phylogenetic trees were constructed in MEGA v12.1 using the Neighbor-Joining (NJ) and Maximum Likelihood (ML) methods with 1000 bootstrap replicates [18]. The resulting trees were visualized using iTOL v6 [19]. Nucleotide identity was calculated using MegAlign v5.03 (DNASTAR, Inc., Madison, WI, USA). Homologous *cox2* sequences from *Pythium rhizo-oryzae* B. Paul (LC553641) and *Pythium catenulatum* V.D. Matthews (LC553642) were used as outgroups.

## 3. Results

### 3.1. Compilation of Pythium insidiosum Mitochondrial Cytochrome c Oxidase Subunit II Gene (cox2) Sequences

A comprehensive dataset of *P. insidiosum cox2* sequences was assembled from the NCBI database and our laboratory culture collection (Supplementary Table S1). Among the NCBI-derived sequences, 210 annotated *cox2* sequences were identified, including 203 from *P. insidiosum* (identity range: 92.2–100.0%) and 7 from *Pythium periculosum* (95.1–100.0%). Of these, 18 sequences showed less than 98.5% identity to the reference sequences. The broader NCBI search identified an additional 78 sequences meeting the inclusion criterion of ≥98.5% sequence identity, including 76 *P. insidiosum*, one *P. periculosum*, and one *Lagenidium* sp. After applying the selection criteria described above, a total of 211 sequences were retained from NCBI. To further expand dataset coverage, 78 *cox2* sequences generated from *P. insidiosum* isolates in the Ramathibodi Hospital Culture Collection were included in the analysis (GenBank accession numbers LC805689-LC805766). After removal of 40 duplicate sequences from 38 isolates based on sequence length and gap content, the final dataset comprised 249 unique sequences, including 171 from NCBI and 78 from our laboratory collection (Supplementary Table S1). This curated dataset provides a robust foundation for downstream analyses of genetic diversity, genotyping, and phylogenetic relationships.

### 3.2. Multiple Sequence Alignment Analysis

A total of 249 *cox2* sequences, together with two outgroup species (*Pythium rhizo-oryzae* and *Pythium catenulatum*), were aligned using the MUSCLE and BMGE algorithms. The resulting sequences ranged from 435 to 663 bp in length. To standardize alignment length, sequences were trimmed to a uniform 387-bp region corresponding to nucleotide positions 182–568 of the complete 771-bp *cox2* coding sequence (accession YP_009167017) [20]. Within this trimmed region, 18 single-nucleotide polymorphisms (SNPs) were identified, allowing classification of the dataset into four distinct groups (Table 1). Analysis of the SNP profiles revealed clear nucleotide differences among the major clades. Several SNP positions were clade-specific and contributed to the differentiation of Clades I–IV. For example, Clade I was distinguished by informative nucleotides at positions 213 and 408, Clade II possessed characteristic nucleotides at positions 297 and 336, Clade III was characterized by the nucleotide at position 369, and Clade IV displayed a unique nucleotide at position 360. Collectively, these 18 SNPs formed an informative nucleotide signature that supported genotype assignment and phylogenetic classification.

### 3.3. Geographic, Species, and Host Distributions

**Figure 1:**
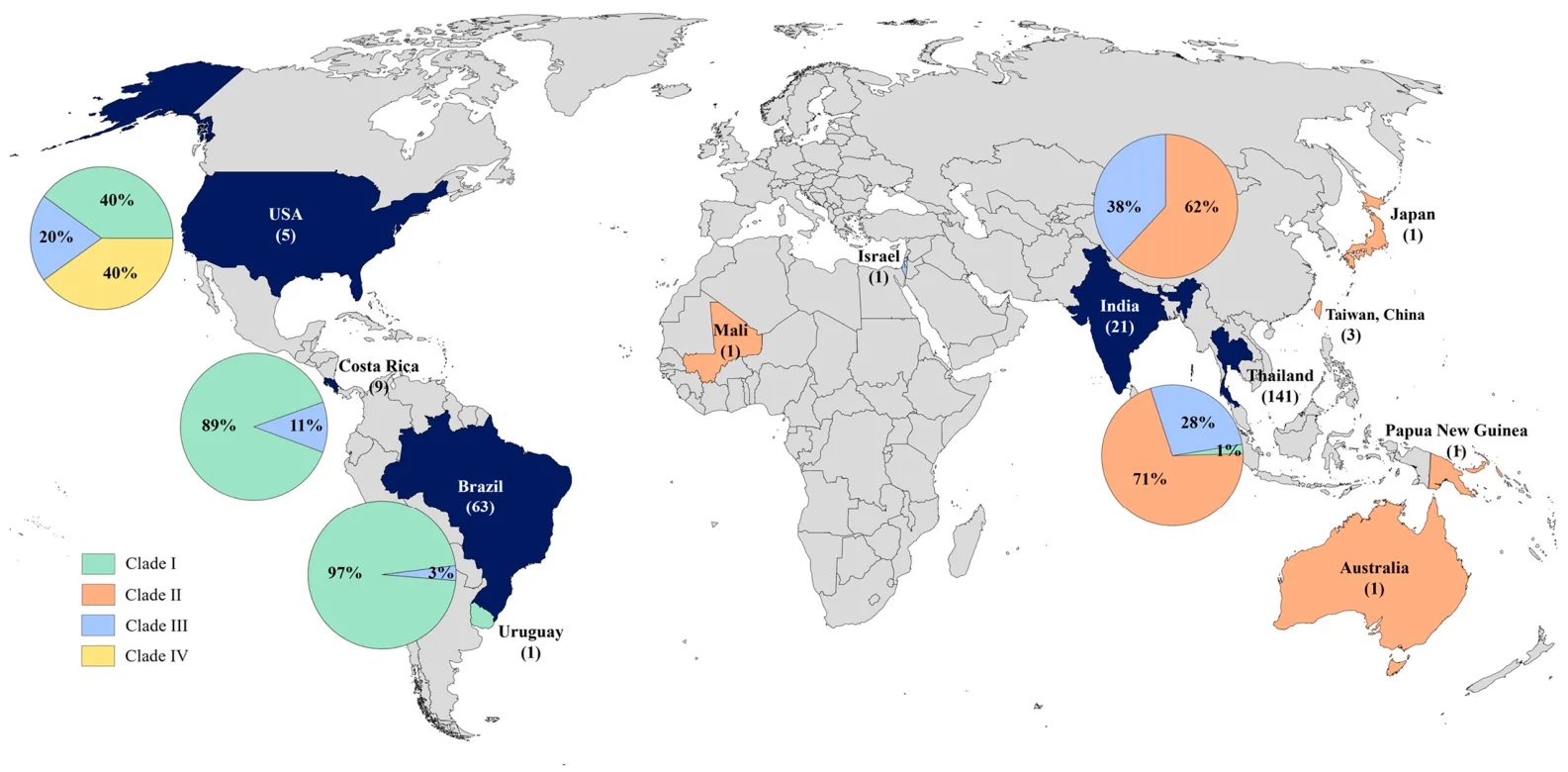
**Geographic origins and genotype distribution of *Pythium insidiosum* isolates included in the present study.** Geographic origins of *P. insidiosum* isolates included in this study (*n* = 249), showing the distribution of genotypes among the analyzed isolates. Numbers in parentheses indicate the number of mitochondrial cytochrome c oxidase subunit II gene (*cox2*) sequences from each country included in the phylogenetic analysis. Countries are color-coded by reported *cox2* genotypes: Clade I (green), Clade II (orange), Clade III (purple), and Clade IV (yellow). The United States, Costa Rica, Brazil, India, and Thailand are highlighted in dark blue to indicate the co-circulation of multiple genotypes of *P. insidiosum* and closely related species (*Pythium periculosum*). Pie charts represent the proportional distribution of clades (I–IV), with slice sizes corresponding to genotype frequencies (%). The map was generated in Microsoft Excel 2021 using metadata compiled from NCBI and laboratory-derived sequences. The map represents the geographic origins of the isolates analyzed in this study and not the complete global distribution of *P. insidiosum*.

Of the 249 sequences included in the analysis, 241 (96.8%) were derived from *P. insidiosum*, 7 (2.8%) from *P. periculosum*, and one (0.4%) from a *Lagenidium* sp. Geographically, most sequences originated from Thailand (56.6%, *n* = 141), followed by Brazil (25.3%, *n* = 63) and India (8.4%, *n* = 21) (Figure 1). Together, these three countries accounted for 90.4% of all sequences included in the dataset. The remaining sequences (9.6%) were distributed across multiple countries, including Costa Rica (4.0%, *n* = 10), the United States (2.0%, *n* = 5), Taiwan, China (1.2%, *n* = 3), and single entries from Uruguay, Mali, Israel, Japan, Papua New Guinea, and Australia (Supplementary Table S1). Host distribution analysis showed that human-derived isolates were most common (43.0%, *n* = 107), followed by equine hosts (28.5%, *n* = 71) and canine hosts (3.6%, *n* = 9). Other sources included mosquito larvae (1.6%, *n* = 4) and a bear (0.4%, *n* = 1), while six isolates (2.4%) had unknown hosts. Environmental samples accounted for 20.5% (*n* = 51) of the dataset, representing the third largest source category after human and equine hosts. These findings demonstrate broad host representation within the dataset and highlight the substantial representation of environmental isolates within the analyzed sequence collection.

### 3.4. Phylogenetic and Subclade Analyses

**Figure 2:**
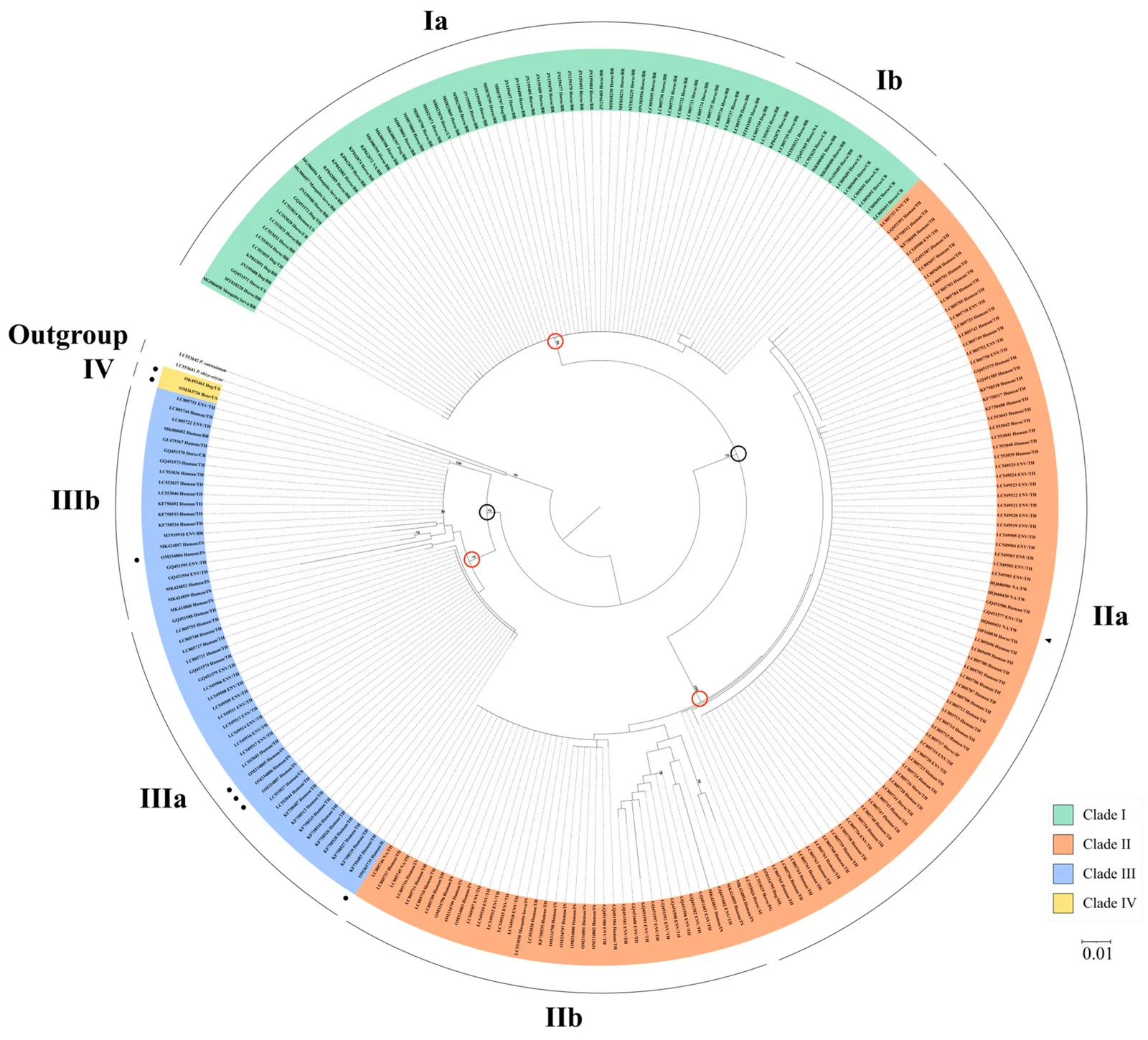
**Maximum Likelihood phylogenetic relationships of *Pythium insidiosum* inferred from partial mitochondrial cytochrome c oxidase subunit II gene (*cox2*) sequences.** A circular phylogenetic tree was constructed from 249 *Pythium* isolates based on a 387-bp region of the mitochondrial *cox2* gene. The dataset includes *P. insidiosum*, *Pythium periculosum* (indicated by black dots), and one *Lagenidium* sp. (indicated by an arrowhead), with *Pythium rhizo-oryzae* and *Pythium catenulatum* used as outgroups. Black circles indicate nodes supported by bootstrap values ≥ 70% that define the major clades (I–IV), whereas red circles indicate nodes defining subclades (Ia, Ib, IIa, IIb, IIIa, and IIIb). Four major clades are highlighted by distinct colors: Clade I (green), Clade II (orange), Clade III (purple), and Clade IV (yellow). Bootstrap values (≥70%) are shown at branch nodes. Subclades within Clades I–III are defined according to clustering patterns. Isolates are labeled with an accession number and the host or source. A high-resolution rectangular (phylogram) version of the tree is provided as Supplementary Figure S2.

Phylogenetic analysis of the 249 isolates (including one *Lagenidium* sp.) and two outgroup species was conducted using the *cox2* gene. The resulting ML tree revealed four major clades (I–IV) with uneven distribution (Figure 2). Clade II was the largest (48.2%, *n* = 120), representing nearly half of all analyzed isolates and exceeding the combined size of Clades III (20.9%, *n* = 52) and IV (0.8%, *n* = 2). Clade I accounted for 30.1% (*n* = 75) of the dataset, whereas Clade IV was represented by only two isolates (0.8%). With a bootstrap cutoff of ≥70%, six subclades were identified within Clades I–III (Figure 2; Supplementary Table S1). Subclade IIa was the most abundant (32.9%, *n* = 82), followed by Ia (24.1%, *n* = 60), IIb (15.3%, *n* = 38), IIIa (12.4%, *n* = 31), IIIb (8.4%, *n* = 21), and Ib (6.0%, *n* = 15). The NJ phylogeny (Supplementary Figure S1) recovered the same major clades and subclades as the ML analysis, supporting the robustness and consistency of the inferred phylogenetic structure.

Sequence identity analysis indicated high conservation within clades. A total of 57.1% (*n* = 142) of the isolates showed complete sequence identity to the reference sequences (Supplementary Table S1), whereas 35.7% (*n* = 89) exhibited minor variation (99.0–99.8%). Lower identity values (<98.5%) were observed in 7.2% of the isolates (*n* = 18; nucleotide identity range: 94.4–98.4%), with most of these isolates belonging to subclades IIb (*n* = 11, 61.1%) and IIIb (*n* = 7, 38.9%). Intra-clade nucleotide identity ranged from 99.2–100.0% in Clade I, 93.0–100.0% in Clade II, 92.0–100.0% in Clade III, and 100.0% in Clade IV (Table 2). Inter-clade nucleotide identity was consistently lower, ranging from 88.4% to 97.4%, supporting genetic separation among the major clades.

## 4. Discussion

### 4.1. Comprehensive Mitochondrial Cytochrome c Oxidase Subunit II Gene (cox2) Dataset and Methodological Framework

This study represents an advancement in the molecular characterization of *Pythium insidiosum* and related species by compiling a comprehensive *cox2* dataset (249 unique sequences) through the integration of 171 publicly available *cox2* sequences from NCBI with 78 newly generated sequences. This effort establishes a robust global reference dataset encompassing diverse hosts, geographic regions, clades, and closely related taxa. Strict data curation, including application of a ≥98.5% identity threshold and removal of duplicate and low-quality sequences, ensured analytical reliability and reproducibility. In addition, the use of a standardized 387-bp *cox2* fragment (positions 182–568), combined with consistent alignment, trimming, and bootstrap-supported phylogenetic inference (≥70%), enables reproducible comparative analyses across studies. In *P. insidiosum*, the *cox2* gene was selected because previous studies have demonstrated its utility for molecular identification, phylogenetic analysis, and genotyping [4,14]. Its single-copy nature simplifies alignment while retaining sufficient variation for phylogenetic inference [21].

Given that *P. insidiosum* is a life-threatening oomycete responsible for severe infections in humans and animals, particularly in tropical and subtropical regions with high freshwater exposure [7], such a comprehensive dataset provides a valuable genetic resource for future studies of *P. insidiosum*. Although the current geographic distribution reflects uneven sampling (e.g., dominance of sequences from Thailand and Brazil), this study provides a methodological foundation for future global sampling and supports expanded international collaboration.

### 4.2. Phylogenetic Insights and Applications

Phylogenetic analysis based on the standardized 387-bp *cox2* fragment identified four major phylogenetic clades (I–IV), consistent with previous ribosomal DNA internal transcribed spacer region (ITS)-based classifications [8], while additionally resolving six well-supported subclades (Ia, Ib, IIa, IIb, IIIa, and IIIb). The NJ and ML phylogenetic analyses recovered the same major clades and subclades (Figure 2 and Supplementary Figure S1), indicating that the inferred phylogenetic structure was robust to the tree-building method employed. These findings highlight genetic complexity and support the concept that *P. insidiosum* represents a species complex rather than a homogeneous taxon.

The major clades recovered in the present *cox2*-based phylogeny are generally consistent with those reported in previous studies of *P. insidiosum* [8,14]. At present, direct comparisons between *cox1*- and *cox2*-based phylogenies of a large and geographically diverse collection of *P. insidiosum* isolates remain limited. Therefore, although the available evidence suggests general concordance among commonly used molecular markers, further multilocus studies would be valuable for evaluating phylogenetic congruence and refining our understanding of genetic relationships within the species complex.

The identification of an 18-nucleotide signature motif composed of informative SNPs within the 387-bp *cox2* fragment enabled differentiation among clades, providing both phylogenetic resolution and practical markers for genotyping. While the *cox2* gene is relatively conserved due to its essential role in cellular respiration [22], it retains sufficient variability for lineage discrimination, providing an effective balance between sequence conservation and phylogenetic resolution [4,21]. This is supported by high intra-clade identity coupled with lower inter-clade identity (Table 2), indicating strong genetic partitioning. Sequences with ≥98.5% identity generally correspond to the same lineage [15], whereas those below this threshold may still belong to the same species but reflect deeper divergence, consistent with fungal barcoding principles [23,24]. The presence of complete sequence identity in many isolates suggests recent divergence or strong purifying selection, while sequences below 98.5% indicate ongoing diversification and underexplored genetic variation.

Importantly, the identification of these SNP markers enhances the utility of *cox2* by enabling rapid genotype assignment without requiring full phylogenetic reconstruction. This supports scalable analyses and provides lineage-informative markers that may serve as a foundation for future molecular assay development. If developed and validated, such assays could facilitate rapid lineage assignment and studies of genetic diversity. However, the clinical significance of the currently recognized clades remains unclear, and further studies are needed to determine whether genetic lineages differ in characteristics such as geographic distribution, host range, virulence, environmental adaptation, or treatment response. Collectively, these features position *cox2* as a phylogenetic marker and a tool for studies of genetic diversity and lineage assignment in *P. insidiosum*.

### 4.3. Taxonomic Framework and Species Relationships

This study established a quantitative taxonomic framework for *P. insidiosum* and related species by defining inter-clade identity ranges and evaluating phylogenetic relationships among a large collection of isolates. The previously proposed 98.5% *cox2* identity threshold was used as a practical guideline for sequence screening and species-level identification, while acknowledging that sequence identity cutoffs are not universally applicable across all taxa [23,24]. Several isolates included in the present study exhibited nucleotide identity values below 95% relative to the selected reference sequences while remaining annotated as *P. insidiosum* in public databases and clustering within recognized *P. insidiosum* phylogenetic lineages. These isolates were retained because highly divergent sequences may provide valuable information regarding genetic diversity and evolutionary relationships within the species complex. Nevertheless, such isolates should be interpreted with caution, as some may represent deeply divergent *P. insidiosum* lineages, taxonomic misidentifications, or potentially undescribed species. In addition, errors in public database annotations cannot be excluded. Because species boundaries cannot be established on the basis of a single genetic marker alone, additional evidence from multilocus phylogenetic analyses, morphological characterization, and genome-scale investigations will be necessary to resolve the taxonomic status of these isolates. The phylogenetic placement of related taxa further strengthens the robustness of this framework. *Pythium periculosum* formed a distinct lineage [8], primarily within Clades III and IV, supporting its taxonomic separation.

The occurrence of both *P. insidiosum* and *P. periculosum* annotations within Clades III and IV should be interpreted in the context of the taxonomic revision proposed by Miraglia et al. [8]. Because many *cox2* sequences currently available in GenBank were deposited before the recognition of *P. periculosum* as a distinct taxon, numerous sequences belonging to these lineages remain annotated as *P. insidiosum*. Consequently, the high sequence identity observed between isolates carrying the two species designations reflects historical annotation practices in public databases rather than a contradiction of the proposed taxonomic framework. At present, the available *cox2* data alone do not allow definitive assignment of all Clade III isolates to either *P. insidiosum* or *P. periculosum*. Consequently, the possibility that some Clade III isolates represent historically misannotated *P. periculosum* sequences cannot be excluded. Likewise, the currently available data are insufficient to determine whether *P. periculosum* should be restricted exclusively to Clade IV. Additional multilocus phylogenetic analyses and genome-scale studies will be necessary to refine species boundaries within these closely related lineages.

In contrast, the placement of a sequence annotated as *Lagenidium* sp. within subclade IIa suggests a possible misidentification or annotation error in the public database, although additional studies would be required to conclusively determine its taxonomic status. This observation highlights an important limitation of publicly available sequence repositories, where taxonomic assignments may occasionally remain unverified or be based on incomplete morphological or molecular evidence. Consequently, researchers should carefully evaluate sequence identity, phylogenetic placement, and associated metadata before incorporating public sequences into comparative analyses. Given that morphology alone may not reliably distinguish closely related oomycetes, further investigation of such isolates should include detailed morphological and physiological characterization, along with additional molecular marker analyses. Such integrative analyses would help confirm taxonomic identity, detect potential errors in database annotations, and improve the accuracy of future phylogenetic and epidemiological studies.

Although the major clades and subclades identified in *P. insidiosum* are well supported by molecular phylogenetic analyses, corresponding morphological and physiological differences among these groups remain poorly characterized. At present, no systematic studies have evaluated whether the genetic lineages defined by molecular markers are associated with differences in morphology, growth characteristics, environmental adaptation, host range, pathogenicity, or other biologically relevant traits. Future investigations integrating phylogenetic, morphological, physiological, ecological, and pathogenicity data may provide important insights into the biological significance of the observed genetic diversity and further refine our understanding of evolutionary relationships within the *P. insidiosum* species complex.

These findings emphasize the importance of careful sequence validation and highlight the need for integrative taxonomic approaches combining molecular, morphological, and ecological evidence, particularly for sequences near taxonomic boundaries. Outliers or borderline sequences should not be viewed as limitations but rather as evidence of dynamic evolutionary processes and potential cryptic diversity within the *P. insidiosum* complex, providing opportunities for future multi-gene and genome-scale analyses.

### 4.4. Ecological and Host-Associated Observations

This study documented ecological diversity in *P. insidiosum*, demonstrating a broad host spectrum that includes humans, horses, other mammals, and environmental reservoirs. Phylogenetic, geographic, and host-associated patterns were observed within the currently available *cox2* dataset. Within this dataset, Clade I was most frequently represented by animal-derived isolates from the Americas, particularly horses, whereas Clades II and III were most frequently represented by human-derived isolates from Asia, particularly Thailand. Clade II has also been represented by isolates from Africa and Australia, although less frequently.

Approximately 98% of the environmental isolates included in this study originated from Thailand. This marked imbalance likely reflects substantial sampling bias resulting from the limited availability of environmental *P. insidiosum* isolates from regions outside Thailand. Consequently, the observed geographic distribution should be interpreted with caution and may not accurately reflect the pathogen’s global environmental occurrence. Additional environmental sampling from underrepresented regions will be essential to better define the worldwide distribution and ecological diversity of *P. insidiosum*.

Although pythiosis cases have been reported from mainland China, including a recent study describing human pythiosis and a Chinese *P. insidiosum* isolate [25], no corresponding *cox2* sequence data from Chinese isolates were publicly available at the time of data collection for the present study. Consequently, isolates from mainland China could not be included in the present phylogenetic analysis. This limitation highlights the dependence of sequence-based comparative studies on publicly accessible molecular datasets and underscores the need for broader geographic representation of *cox2* sequences from underrepresented regions. Future incorporation of isolates from mainland China and other incompletely sampled areas may provide a more comprehensive understanding of the genetic diversity and global distribution of *P. insidiosum*.

Similarly, the apparent predominance of Clade II among Asian isolates and Clade I among isolates from the Americas should be regarded as observations within the currently available dataset rather than evidence of statistically supported biogeographic structure, because substantial geographic sampling imbalances exist among regions. Future studies using more geographically balanced collections will be necessary to determine whether these patterns reflect true population structure or are primarily the result of sampling bias. An interesting observation was that a few Clade I isolates have been isolated from dogs in Thailand [26], despite Clades II and III being predominantly reported as the causative agents of pythiosis in this region (Figure 1; Supplementary Table S1). Conversely, a small number of Clade III isolates were identified in human, horse, and environmental samples in the Americas (the United States, Costa Rica, and Brazil), representing atypical host and geographic distributions for this genotype (Figure 1; Supplementary Table S1).

The presence of multiple clades in the currently available *cox2* dataset indicates substantial genetic diversity within the species complex, although the full extent of global diversity remains uncertain due to uneven geographic sampling. However, previous studies have suggested that ecological, environmental, climatic, and host-related factors may influence infection patterns and susceptibility. For example, the high prevalence of thalassemia in Asia has been associated with human pythiosis, supporting the role of host predisposing conditions in disease occurrence [27]. The close phylogenetic relationship between clinical and environmental isolates is consistent with the hypothesis that environmental reservoirs may contribute to human and animal exposure. Together with previous studies, these observations underscore the potential importance of environmental factors, including water systems and climate, in the ecology and distribution of *P. insidiosum* [28,29,30].

## 5. Conclusions

This study provides a comprehensive mitochondrial cytochrome c oxidase subunit II gene (*cox2*)-based framework for investigating the genetic diversity of *Pythium insidiosum* and related species. The integration of a large, high-quality sequence dataset with robust phylogenetic analysis provides a reliable foundation for species identification and genotyping. The use of *cox2* as a molecular marker demonstrated discriminatory power and practical utility for lineage assignment and may support future development of molecular diagnostic tools. The availability of a well-curated reference dataset further enhances the reliability of sequence-based identification, including NCBI BLAST analyses, and facilitates consistent interpretation of *cox2* sequence data across studies. Despite these advances, expanded sampling, particularly from underrepresented clades such as Clade IV, will be essential to further refine classification accuracy and better characterize global genetic diversity. Overall, this work provides a molecular and phylogenetic framework for the study of *P. insidiosum* and establishes a resource for future taxonomic, evolutionary, and phylogenetic investigations.

## Figures and Tables

**Table 1:** Informative nucleotide polymorphisms in the mitochondrial cytochrome c oxidase subunit II gene (*cox2*) defining *Pythium insidiosum* clades. Summary of 18 single-nucleotide polymorphic (SNP) positions identified within a 387-bp region of the *cox2* gene used for genotyping. Nucleotide positions are mapped relative to the reference *cox2* coding sequence (YP_009167017). Distinct SNP patterns define the four major clades (I–IV) of *P. insidiosum*, distinguish the related species (*Pythium periculosum*), and differentiate the outgroup species (*Pythium catenulatum* and *Pythium rhizo-oryzae*). Clade-specific nucleotides are indicated in brackets.

Species & Clade (Number of Isolates)	Nucleotide Position of *cox2* Gene																	
	213	225	237	279	282	291	294	297	324	333	336	360	369	408	489	537	546	562
*P. insidiosum* Clade I (*n* = 75)	[A]	T	A	A	A	A	T	T	T	T	T	T	T	[C]	T	A	T	A
*P. insidiosum* Clade II (*n* = 120)	T	C	C	A	G	T	T	[C]	T	T	[A]	T	T	T	C	A	T	G
Clade III * (*n* = 52)	T	T	A	T	A	T	A	T	A	A	T	T	[C]	T	T	T	A	G
*P. periculosum* Clade IV (*n* = 2)	T	T	T	C	A	A	A	T	A	A	T	[C]	T	T	T	T	A	G
*P. catenulatum* (*n* = 1)	T	T	C	A	G	A	A	T	C	A	T	T	T	T	C	T	A	A
*P. rhizo-oryzae* (*n* = 1)	T	C	T	T	A	A	A	T	T	T	T	T	T	T	T	T	T	A

Footnote: * Clade III contains isolates currently annotated as both *P. insidiosum* and *P. periculosum* in public databases. The coexistence of these annotations likely reflects historical taxonomic assignments and database records deposited before recognition of *P. periculosum* as a distinct species [8].

**Table 2:** Intra- and inter-clade nucleotide identity of *Pythium insidiosum* mitochondrial cytochrome c oxidase subunit II gene (*cox2*) sequences. Pairwise nucleotide identity (%) among *cox2* sequences within and between clades of *P. insidiosum*. Values are presented as ranges to reflect variability within the dataset.

Clade	Nucleotide Identity (%)			
	I	II	III	IV
I	99.2–100	91.2–96.6	91.0–95.9	95.1–95.6
II	91.2–96.6	93.0–100	88.4–95.3	90.4–94.6
III	91.0–95.9	88.4–95.3	92.0–100	93.3–97.4
IV	95.1–95.6	90.4–94.6	93.3–97.4	100

## Data Availability

All data generated and analyzed in this study are described within the manuscript, and no additional or unpublished datasets are associated with this work.

## Supplementary Materials

The following supporting information can be downloaded at https://www.mdpi.com/article/10.3390/microorganisms14102299/s1: Table S1: Comprehensive list of *Pythium* isolates included in the phylogenetic analysis; Figure S1: Neighbor-Joining phylogenetic relationships of *Pythium insidiosum* inferred from partial mitochondrial cytochrome c oxidase subunit II gene (*cox2*) sequences. Legend details are the same as those described for Figure 2. A high-resolution rectangular (phylogram) version of the tree is provided as Supplementary Figure S3. Figure S2: Maximum Likelihood high-resolution rectangular phylogenetic tree of *Pythium insidiosum* inferred from partial mitochondrial cytochrome c oxidase subunit II gene (*cox2*) sequences. This figure represents the rectangular version of Figure 2. Figure S3: Neighbor-Joining high-resolution rectangular phylogenetic tree of *Pythium insidiosum* inferred from partial mitochondrial cytochrome c oxidase subunit II gene (*cox2*) sequences. This figure represents the rectangular version of Supplementary Figure S1.

## References

[1] Masigol H., Solbach M.D., Pourmoghaddam M.J., Ahadi R., Mostowfizadeh-Ghalamfarsa R., Taheri S.R., Tobias-Hünefeldt S.P., Bonkowski M., Grossart H.-P. (2025). A Glimpse into Oomycota Diversity in Freshwater Lakes and Adjacent Forests Using a Metabarcoding Approach. Sci. Rep..

[2] Gaastra W., Lipman L.J.A., De Cock A.W.A.M., Exel T.K., Pegge R.B.G., Scheurwater J., Vilela R., Mendoza L. (2010). *Pythium insidiosum*: An Overview. Vet. Microbiol..

[3] Mendoza L., Kaufman L., Standard P. (1987). Antigenic Relationship between the Animal and Human Pathogen *Pythium insidiosum* and Nonpathogenic *Pythium* Species. J. Clin. Microbiol..

[4] Mar Htun Z., Laikul A., Pathomsakulwong W., Yurayart C., Lohnoo T., Yingyong W., Kumsang Y., Payattikul P., Sae-Chew P., Rujirawat T. (2021). Identification and Biotyping of *Pythium insidiosum* Isolated from Urban and Rural Areas of Thailand by Multiplex PCR, DNA Barcode, and Proteomic Analyses. J. Fungi.

[5] Zambrano C.G., Fonseca A.O.S., Valente J.S.S., Braga C.Q., Sallis E.S.V., Azevedo M.I., Weiblen C., Santurio J.M., Botton S.A., Pereira D.I.B. (2017). Isolamento e Caracterização de Espécies de *Pythium* de Ambientes Aquáticos No Estado Do Rio Grande Do Sul e Avaliação Da Patogenicidade Em Modelo Experimental. Pesqui. Vet. Bras..

[6] Medhasi S., Chindamporn A., Worasilchai N. (2022). A Review: Antimicrobial Therapy for Human Pythiosis. Antibiotics.

[7] Yolanda H., Krajaejun T. (2022). Global Distribution and Clinical Features of Pythiosis in Humans and Animals. J. Fungi.

[8] Miraglia B.M., Mendoza L., Rammohan R., Vilela L., Vilela C., Vilela G., Huebner M., Mani R., Vilela R. (2022). *Pythium insidiosum* Complex Hides a Cryptic Novel Species: *Pythium periculosum*. Fungal Biol..

[9] Presser J.W., Goss E.M. (2015). Environmental Sampling Reveals That *Pythium insidiosum* Is Ubiquitous and Genetically Diverse in North Central Florida. Med. Mycol..

[10] Xu J. (2016). Fungal DNA Barcoding. Genome.

[11] Schurko A.M., Mendoza L., Lévesque C.A., Désaulniers N.L., de Cock A.W.A.M., Klassen G.R. (2003). A Molecular Phylogeny of *Pythium insidiosum*. Mycol. Res..

[12] Robideau G.P., De Cock A.W.A.M., Coffey M.D., Voglmayr H., Brouwer H., Bala K., Chitty D.W., Dé-saulniers N., Eggertson Q.A., Gachon C.M.M. (2011). DNA Barcoding of Oomycetes with Cytochrome c Oxidase Subunit I and Internal Transcribed Spacer. Mol. Ecol. Resour..

[13] Appavu S.P., Prajna L., Rajapandian S.G.K. (2020). Genotyping and Phylogenetic Analysis of *Pythium insidiosum* Causing Human Corneal Ulcer. Med. Mycol..

[14] Kammarnjesadakul P., Palaga T., Sritunyalucksana K., Mendoza L., Krajaejun T., Vanittanakom N., Tongchusak S., Denduangboripant J., Chindamporn A. (2011). Phylogenetic Analysis of *Pythium insidiosum* Thai Strains Using Cytochrome Oxidase II (COX II) DNA Coding Sequences and Internal Transcribed Spacer Regions (ITS). Med. Mycol..

[15] Mahadevakumar S., Sridhar K.R., Rai M., Abd-Elsalam K.A., Ingle A.P. (2020). Diagnosis of Pythium by Classical and Molecular Approaches. Pythium.

[16] Lohnoo T., Jongruja N., Rujirawat T., Yingyon W., Lerksuthirat T., Nampoon U., Kumsang Y., Onpaew P., Chongtrakool P., Keeratijarut A. (2014). Efficiency Comparison of Three Methods for Extracting Genomic DNA of the Pathogenic Oomycete *Pythium insidiosum*. J. Med. Assoc. Thai..

[17] Lemoine F., Correia D., Lefort V., Doppelt-Azeroual O., Mareuil F., Cohen-Boulakia S., Gascuel O. (2019). NGPhylogeny.Fr: New Generation Phylogenetic Services for Non-Specialists. Nucleic Acids Res..

[18] Stecher G., Suleski M., Tao Q., Tamura K., Kumar S. (2026). MEGA 12.1: Cross-Platform Release for macOS and Linux Operating Systems. J. Mol. Evol..

[19] Letunic I., Bork P. (2021). Interactive Tree Of Life (iTOL) v5: An Online Tool for Phylogenetic Tree Display and An-notation. Nucleic Acids Res..

[20] Tangphatsornruang S., Ruang-Areerate P., Sangsrakru D., Rujirawat T., Lohnoo T., Kittichotirat W., Pa-tumcharoenpol P., Grenville-Briggs L.J., Krajaejun T. (2016). Comparative Mitochondrial Genome Analysis of *Pythium insidiosum* and Related Oomycete Species Provides New Insights into Genetic Variation and Phylogenetic Relationships. Gene.

[21] Li Y., Hu X.-D., Yang R.-H., Hsiang T., Wang K., Liang D.-Q., Liang F., Cao D.-M., Zhou F., Wen G. (2015). Complete Mitochondrial Genome of the Medicinal Fungus *Ophiocordyceps sinensis*. Sci. Rep..

[22] Choi Y.-J., Beakes G., Glockling S., Kruse J., Nam B., Nigrelli L., Ploch S., Shin H.-D., Shivas R.G., Telle S. (2015). Towards a Universal Barcode of Oomycetes—A Comparison of the Cox1 and Cox2 Loci. Mol. Ecol. Resour..

[23] Garnica S., Schön M.E., Abarenkov K., Riess K., Liimatainen K., Niskanen T., Dima B., Soop K., Frøslev T.G., Jeppesen T.S. (2016). Determining Threshold Values for Barcoding Fungi: Lessons from *Cortinarius* (Ba-sidiomycota), a Highly Diverse and Widespread Ectomycorrhizal Genus. FEMS Microbiol. Ecol..

[24] Lücking R., Aime M.C., Robbertse B., Miller A.N., Ariyawansa H.A., Aoki T., Cardinali G., Crous P.W., Druzhinina I.S., Geiser D.M. (2020). Unambiguous Identification of Fungi: Where Do We Stand and How Ac-curate and Precise Is Fungal DNA Barcoding?. IMA Fungus.

[25] Liu X., Diao Q., Li M., Yang Y., Liang J., Cui Z., Zhang H., He H., Huang J., Ren H. (2025). Worldwide Distribution of Human Pythosis and Biological Characteristics of a *Pythium insidiosum* Strain Susceptible to Antibiotics from China: A Dual-Scale Study. Front. Med..

[26] Chindamporn A., Kammarnjessadakul P., Kesdangsakonwut S., Banlunara W. (2020). A Case of Canine Cutaneous Pythiosis in Thailand. Access Microbiol..

[27] Krajaejun T., Sathapatayavongs B., Pracharktam R., Nitiyanant P., Leelachaikul P., Wanachiwanawin W., Chaiprasert A., Assanasen P., Saipetch M., Mootsikapun P. (2006). Clinical and Epidemiological Analyses of Human Pythiosis in Thailand. Clin. Infect. Dis..

[28] Jara M., Holcomb K., Wang X., Goss E.M., Machado G. (2021). The Potential Distribution of *Pythium insidiosum* in the Chincoteague National Wildlife Refuge, Virginia. Front. Vet. Sci..

[29] Machado G., Weiblen C., Escobar L.E. (2018). Potential Distribution of *Pythium insidiosum* in Rio Grande Do Sul, Brazil, and Projections to Neighbour Countries. Transbound. Emerg. Dis..

[30] Yin M.S., Meth P., Haddawy P., Bicout D.J., Krajaejun T. (2026). Mapping Environmental Suitability for *Pythium insidiosum* under Current and Future Climate Conditions. iScience.

