# Supplementary material for: A Comprehensive Mitochondrial Cytochrome c Oxidase Subunit II Gene (cox2)-Based Framework for Genotyping and Phylogenetic Analysis of Pythium insidiosum and Related Taxa
Source: Microorganisms. 2026 Oct 9;14(10):2299. doi: 10.3390/microorganisms14102299 (PMC13707720; doi:10.3390/microorganisms14102299)
Supplement: Supplementary file 1 [file microorganisms-14-02299-s001.zip › Supplementary Figure S2 (ML reg).pdf]

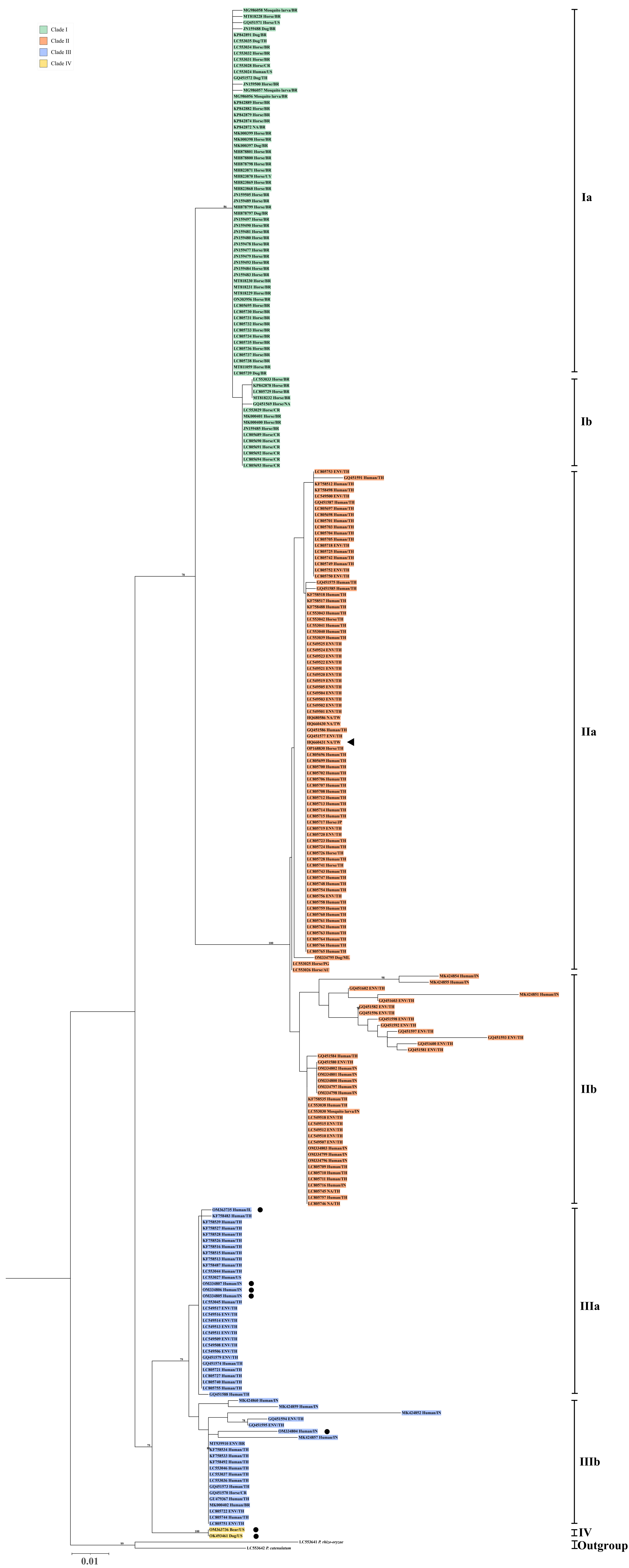

Maximum-likelihood (ML) algorithm
