# Supplementary material for: A Comprehensive Mitochondrial Cytochrome c Oxidase Subunit II Gene (cox2)-Based Framework for Genotyping and Phylogenetic Analysis of Pythium insidiosum and Related Taxa
Source: Microorganisms. 2026 Oct 9;14(10):2299. doi: 10.3390/microorganisms14102299 (PMC13707720; doi:10.3390/microorganisms14102299)
Supplement: Supplementary file 1 [file microorganisms-14-02299-s001.zip › Supplementary Table S1.pdf]

Supplementary Table S1. Comprehensive list of *Pythium* isolates included in the phylogenetic analysis.

Number	Species	Strain	Source	Country	Accession number	Duplicate sequences	% Nucleotide identity	Genotype	Sub-clade	Sequence Source
1	*P. insidiosum*	Pi009C1	Horse	Brazil	LC805695		Reference	Clade I	Ia	This study
2	*P. insidiosum*	295	Dog	Brazil	MK000397		100	Clade I	Ia	GenBank
3	*P. insidiosum*	Can-1	Dog	Brazil	KP842891		100	Clade I	Ia	GenBank
4	*P. insidiosum*	Pi073C1	Dog	Brazil	LC805739		100	Clade I	Ia	This study
5	*P. insidiosum*	Lapemi 293	Dog	Brazil	MH878797		100	Clade I	Ia	GenBank
6	*P. insidiosum*	LAPEMI126	Horse	Brazil	JN159483		100	Clade I	Ia	GenBank
7	*P. insidiosum*	337	Horse	Brazil	MK000399		100	Clade I	Ia	GenBank
8	*P. insidiosum*	296	Horse	Brazil	MK000398		100	Clade I	Ia	GenBank
9	*P. insidiosum*	Lapemi 152	Horse	Brazil	MH878801		100	Clade I	Ia	GenBank
10	*P. insidiosum*	Lapemi 254	Horse	Brazil	MH878800		100	Clade I	Ia	GenBank
11	*P. insidiosum*	Lapemi 291	Horse	Brazil	MH878798		100	Clade I	Ia	GenBank
12	*P. insidiosum*	LAPEMI 142	Horse	Brazil	MH823871		100	Clade I	Ia	GenBank
13	*P. insidiosum*	LAPEMI 137	Horse	Brazil	MH823869		100	Clade I	Ia	GenBank
14	*P. insidiosum*	LAPEMI 135	Horse	Brazil	MH823868		100	Clade I	Ia	GenBank
15	*P. insidiosum*	LAPEMI261	Horse	Brazil	JN159505		100	Clade I	Ia	GenBank
16	*P. insidiosum*	LAPEMI148	Horse	Brazil	JN159489		100	Clade I	Ia	GenBank
17	*P. insidiosum*	eq31	Horse	Brazil	MT818231		100	Clade I	Ia	GenBank
18	*P. insidiosum*	EQ30	Horse	Brazil	MT818230		100	Clade I	Ia	GenBank
19	*P. insidiosum*	Eq-29	Horse	Brazil	MT818229		100	Clade I	Ia	GenBank
20	*P. insidiosum*	Pi064C1	Horse	Brazil	LC805730	KP842881	100	Clade I	Ia	This study
21	*P. insidiosum*	Eq27	Horse	Brazil	ON303956		100	Clade I	Ia	GenBank
22	*P. insidiosum*	Pi62	Horse	Brazil	LC553034	KP842877	100	Clade I	Ia	[4]
23	*P. insidiosum*	Pi60	Horse	Brazil	LC553032	KP842875	100	Clade I	Ia	[4]
24	*P. insidiosum*	Pi59	Horse	Brazil	LC553031	KP842873	100	Clade I	Ia	[4]
25	*P. insidiosum*	Eq-22	Horse	Brazil	KP842889		100	Clade I	Ia	GenBank
26	*P. insidiosum*	Eq-11	Horse	Brazil	KP842882		100	Clade I	Ia	GenBank
27	*P. insidiosum*	Eq-8	Horse	Brazil	KP842879		100	Clade I	Ia	GenBank
28	*P. insidiosum*	Eq-3	Horse	Brazil	KP842874		100	Clade I	Ia	GenBank
29	*P. insidiosum*	Pi066C1	Horse	Brazil	LC805732	KP842884	100	Clade I	Ia	This study
30	*P. insidiosum*	Pi067C1	Horse	Brazil	LC805733	KP842885	100	Clade I	Ia	This study
31	*P. insidiosum*	Pi068C1	Horse	Brazil	LC805734	KP842886	100	Clade I	Ia	This study
32	*P. insidiosum*	Pi069C1	Horse	Brazil	LC805735	KP842887	100	Clade I	Ia	This study
33	*P. insidiosum*	Pi070C1	Horse	Brazil	LC805736	KP842888	100	Clade I	Ia	This study
34	*P. insidiosum*	Pi071C1	Horse	Brazil	LC805737	KP842890	100	Clade I	Ia	This study
35	*P. insidiosum*	Pi072C1	Horse	Brazil	LC805738	MT811058	100	Clade I	Ia	This study
36	*P. insidiosum*	Pi065C1	Horse	Brazil	LC805731	KP842883	100	Clade I	Ia	This study
37	*P. insidiosum*	LAPEMI129	Horse	Brazil	JN159484		100	Clade I	Ia	GenBank
38	*P. insidiosum*	LAPEMI187	Horse	Brazil	JN159493		100	Clade I	Ia	GenBank
39	*P. insidiosum*	Lapemi 290	Horse	Brazil	MH878799		100	Clade I	Ia	GenBank
40	*P. insidiosum*	LAPEMI124	Horse	Brazil	JN159481		100	Clade I	Ia	GenBank
41	*P. insidiosum*	LAPEMI123	Horse	Brazil	JN159480		100	Clade I	Ia	GenBank
42	*P. insidiosum*	LAPEMI118	Horse	Brazil	JN159478		100	Clade I	Ia	GenBank
43	*P. insidiosum*	LAPEMI156	Horse	Brazil	JN159490		100	Clade I	Ia	GenBank
44	*P. insidiosum*	LAPEMI121	Horse	Brazil	JN159477		100	Clade I	Ia	GenBank
45	*P. insidiosum*	LAPEMI223	Horse	Brazil	JN159497		100	Clade I	Ia	GenBank
46	*P. insidiosum*	LAPEMI119	Horse	Brazil	JN159479		100	Clade I	Ia	GenBank
47	*P. insidiosum*	ARSEF 13235	Mosquito larva	Brazil	MG986056		100	Clade I	Ia	GenBank
48	*P. insidiosum*	B-01	NA	Brazil	KP842872		100	Clade I	Ia	GenBank
49	*P. insidiosum*	Pi07	Horse	Costa Rica	LC553028		100	Clade I	Ia	[4]
50	*P. insidiosum*	PAC2	Dog	Thailand	GQ451572		100	Clade I	Ia	[14]
51	*P. insidiosum*	Pi74	Dog	Thailand	LC553035		100	Clade I	Ia	[4]
52	*P. insidiosum*	0-44.2017	Horse	Uruguay	MH823870		100	Clade I	Ia	GenBank
53	*P. insidiosum*	Pi10	Human	USA	LC553024		100	Clade I	Ia	[4]
54	*P. insidiosum*	Eq-26	Horse	Brazil	MT811059		99.7	Clade I	Ia	GenBank
55	*P. insidiosum*	LAPEMI146	Dog	Brazil	JN159488		99.7	Clade I	Ia	GenBank
56	*P. insidiosum*	LAPEMI245	Horse	Brazil	JN159500		99.7	Clade I	Ia	GenBank
57	*P. insidiosum*	Eq-28	Horse	Brazil	MT818228		99.7	Clade I	Ia	GenBank
58	*P. insidiosum*	ARSEF 13236	Mosquito larva	Brazil	MG986057		99.7	Clade I	Ia	GenBank
59	*P. insidiosum*	ARSEF 13240	Mosquito larva	Brazil	MG986058		99.7	Clade I	Ia	GenBank
60	*P. insidiosum*	MTPI04	Horse	USA	GQ451571		99.7	Clade I	Ia	[14]
61	*P. insidiosum*	LAPEMI136	Horse	Brazil	JN159485		99.7	Clade I	Ib	GenBank
62	*P. insidiosum*	CBS57685	Horse	Brazil	MK000400		99.7	Clade I	Ib	GenBank
63	*P. insidiosum*	CBS57585	Horse	Brazil	MK000401		99.7	Clade I	Ib	GenBank
64	*P. insidiosum*	Pi006C1	Horse	Costa Rica	LC805694	AF196597, KJ595391	99.7	Clade I	Ib	This study
65	*P. insidiosum*	Pi005C1	Horse	Costa Rica	LC805693		99.7	Clade I	Ib	This study
66	*P. insidiosum*	Pi004C1	Horse	Costa Rica	LC805692		99.7	Clade I	Ib	This study
67	*P. insidiosum*	Pi003C1	Horse	Costa Rica	LC805691		99.7	Clade I	Ib	This study
68	*P. insidiosum*	Pi002C1	Horse	Costa Rica	LC805690		99.7	Clade I	Ib	This study
69	*P. insidiosum*	Pi001C1	Horse	Costa Rica	LC805689		99.7	Clade I	Ib	This study
70	*P. insidiosum*	Pi08	Horse	Costa Rica	LC553029	JN159507	99.7	Clade I	Ib	[4]
71	*P. insidiosum*	Eq-7	Horse	Brazil	KP842878		99.5	Clade I	Ib	GenBank

72	*P. insidiosum*	Pi61	Horse	Brazil	LC553033	KP842876	99.5	Clade I	Ib	[4]
73	*P. insidiosum*	eq32	Horse	Brazil	MT818232		99.5	Clade I	Ib	GenBank
74	*P. insidiosum*	MTPI19	Horse	Costa Rica	GQ451569		99.5	Clade I	Ib	[14]
75	*P. insidiosum*	Pi063C1	Horse	Brazil	LC805729	KP842880	99.5	Clade I	Ib	This study
76	*P. insidiosum*	Pi35	Human	Thailand	LC553043	KF758543, NC_027966	Reference	Clade II	IIa	[4]
77	*P. insidiosum*	Pi039C2	Horse	Japan	LC805717		100	Clade II	IIa	This study
78	*P. insidiosum*	CBS 127280	NA	Taiwan, China	HQ680586		100	Clade II	IIa	GenBank
79	*P. insidiosum*	CBS 127279	NA	Taiwan, China	HQ660430		100	Clade II	IIa	GenBank
80	*Lagenidium* sp.	CBS 127276	NA	Taiwan, China	HQ660431		100	Clade II	IIa	GenBank
81	*P. insidiosum*	Pi043C2	Environment	Thailand	LC805720	OP160030	100	Clade II	IIa	This study
82	*P. insidiosum*	CCS05	Environment	Thailand	LC549503		100	Clade II	IIa	[4]
83	*P. insidiosum*	RM9_02	Environment	Thailand	LC549519		100	Clade II	IIa	[4]
84	*P. insidiosum*	RM9_05	Environment	Thailand	LC549522		100	Clade II	IIa	[4]
85	*P. insidiosum*	TRG_02	Environment	Thailand	LC549524		100	Clade II	IIa	[4]
86	*P. insidiosum*	Pi106C2	Environment	Thailand	LC805756		100	Clade II	IIa	This study
87	*P. insidiosum*	TRG_03	Environment	Thailand	LC549525		100	Clade II	IIa	[4]
88	*P. insidiosum*	RM9_04	Environment	Thailand	LC549521		100	Clade II	IIa	[4]
89	*P. insidiosum*	CCS08	Environment	Thailand	LC549505		100	Clade II	IIa	[4]
90	*P. insidiosum*	CCS04	Environment	Thailand	LC549502		100	Clade II	IIa	[4]
91	*P. insidiosum*	RT_02	Environment	Thailand	LC549523		100	Clade II	IIa	[4]
92	*P. insidiosum*	RM9_03	Environment	Thailand	LC549520		100	Clade II	IIa	[4]
93	*P. insidiosum*	CCS07	Environment	Thailand	LC549504		100	Clade II	IIa	[4]
94	*P. insidiosum*	CCS03	Environment	Thailand	LC549501		100	Clade II	IIa	[4]
95	*P. insidiosum*	Pi042C2	Environment	Thailand	LC805719		100	Clade II	IIa	This study
96	*P. insidiosum*	PECM3	Environment	Thailand	GQ451577		100	Clade II	IIa	[14]
97	*P. insidiosum*	Pi056C2	Horse	Thailand	LC805726	OP168828	100	Clade II	IIa	This study
98	*P. insidiosum*	Pi53	Horse	Thailand	LC553042		100	Clade II	IIa	[4]
99	*P. insidiosum*	Pi076C2	Horse	Thailand	LC805741	OP168829	100	Clade II	IIa	This study
100	*P. insidiosum*	KU40020.1	Horse	Thailand	OP168830		100	Clade II	IIa	GenBank
101	*P. insidiosum*	Pi115C2	Human	Thailand	LC805765		100	Clade II	IIa	This study
102	*P. insidiosum*	Pi110C2	Human	Thailand	LC805760		100	Clade II	IIa	This study
103	*P. insidiosum*	Pi098C2	Human	Thailand	LC805748		100	Clade II	IIa	This study
104	*P. insidiosum*	Pi034C2	Human	Thailand	LC805715		100	Clade II	IIa	This study
105	*P. insidiosum*	Pi024C2	Human	Thailand	LC805708	KF758522	100	Clade II	IIa	This study
106	*P. insidiosum*	Pi017C2	Human	Thailand	LC805702		100	Clade II	IIa	This study
107	*P. insidiosum*	Pi011C2	Human	Thailand	LC805696		100	Clade II	IIa	This study
108	*P. insidiosum*	Pi097C2	Human	Thailand	LC805747		100	Clade II	IIa	This study
109	*P. insidiosum*	Pi031C2	Human	Thailand	LC805713		100	Clade II	IIa	This study
110	*P. insidiosum*	Pi116C2	Human	Thailand	LC805766		100	Clade II	IIa	This study
111	*P. insidiosum*	Pi112C2	Human	Thailand	LC805762		100	Clade II	IIa	This study
112	*P. insidiosum*	Pi104C2	Human	Thailand	LC805754		100	Clade II	IIa	This study
113	*P. insidiosum*	PMS1	Human	Thailand	GQ451586		100	Clade II	IIa	[14]
114	*P. insidiosum*	MCC 7	Human	Thailand	KF758488		100	Clade II	IIa	GenBank
115	*P. insidiosum*	Pi32	Human	Thailand	LC553041		100	Clade II	IIa	[4]
116	*P. insidiosum*	Pi014C2	Human	Thailand	LC805699		100	Clade II	IIa	This study
117	*P. insidiosum*	Pi021C2	Human	Thailand	LC805706		100	Clade II	IIa	This study
118	*P. insidiosum*	Pi030C2	Human	Thailand	LC805712		100	Clade II	IIa	This study
119	*P. insidiosum*	Pi052C2	Human	Thailand	LC805723	OP168827	100	Clade II	IIa	This study
120	*P. insidiosum*	Pi108C2	Human	Thailand	LC805758	KF758482	100	Clade II	IIa	This study
121	*P. insidiosum*	Pi113C2	Human	Thailand	LC805763		100	Clade II	IIa	This study
122	*P. insidiosum*	SIMI 820080826	Human	Thailand	KF758517		100	Clade II	IIa	GenBank
123	*P. insidiosum*	Pi111C2	Human	Thailand	LC805761	KF758503	100	Clade II	IIa	This study
124	*P. insidiosum*	Pi058C2	Human	Thailand	LC805728		100	Clade II	IIa	This study
125	*P. insidiosum*	Pi29	Human	Thailand	LC553040		100	Clade II	IIa	[4]
126	*P. insidiosum*	Pi25	Human	Thailand	LC553039		100	Clade II	IIa	[4]
127	*P. insidiosum*	Pi015C2	Human	Thailand	LC805700	KF758508	100	Clade II	IIa	This study
128	*P. insidiosum*	Pi022C2	Human	Thailand	LC805707		100	Clade II	IIa	This study
129	*P. insidiosum*	Pi033C2	Human	Thailand	LC805714		100	Clade II	IIa	This study
130	*P. insidiosum*	Pi054C2	Human	Thailand	LC805724		100	Clade II	IIa	This study
131	*P. insidiosum*	Pi080C2	Human	Thailand	LC805743		100	Clade II	IIa	This study
132	*P. insidiosum*	Pi109C2	Human	Thailand	LC805759	KF758486	100	Clade II	IIa	This study
133	*P. insidiosum*	Pi114C2	Human	Thailand	LC805764		100	Clade II	IIa	This study
134	*P. insidiosum*	KKU 001-2009	Human	Thailand	KF758518		100	Clade II	IIa	GenBank
135	*P. insidiosum*	Pi36	Horse	Australia	LC553026		99.7	Clade II	IIa	[4]
136	*P. insidiosum*	Pi37	Horse	Papua New Guinea	LC553025	AF196596	99.7	Clade II	IIa	[4]
137	*P. insidiosum*	Pi103C2	Environment	Thailand	LC805753		99.7	Clade II	IIa	This study
138	*P. insidiosum*	Pi102C2	Environment	Thailand	LC805752		99.7	Clade II	IIa	This study
139	*P. insidiosum*	Pi100C2	Environment	Thailand	LC805750		99.7	Clade II	IIa	This study
140	*P. insidiosum*	Pi041C2	Environment	Thailand	LC805718		99.7	Clade II	IIa	This study
141	*P. insidiosum*	CCS01	Environment	Thailand	LC549500		99.7	Clade II	IIa	[4]
142	*P. insidiosum*	PC10	Human	Thailand	GQ451575		99.7	Clade II	IIa	[14]
143	*P. insidiosum*	PC6	Human	Thailand	GQ451585		99.7	Clade II	IIa	[14]
144	*P. insidiosum*	PCM1	Human	Thailand	GQ451587		99.7	Clade II	IIa	[14]
145	*P. insidiosum*	SIMI 283-40	Human	Thailand	KF758498		99.7	Clade II	IIa	GenBank
146	*P. insidiosum*	Pi099C2	Human	Thailand	LC805749		99.7	Clade II	IIa	This study
147	*P. insidiosum*	Pi079C2	Human	Thailand	LC805742		99.7	Clade II	IIa	This study
148	*P. insidiosum*	Pi055C2	Human	Thailand	LC805725		99.7	Clade II	IIa	This study
149	*P. insidiosum*	Pi020C2	Human	Thailand	LC805705	KF758495	99.7	Clade II	IIa	This study

150	*P. insidiosum*	Pi019C2	Human	Thailand	LC805704		99.7	Clade II	IIa	This study
151	*P. insidiosum*	Pi018C2	Human	Thailand	LC805703	KF758493	99.7	Clade II	IIa	This study
152	*P. insidiosum*	Pi016C2	Human	Thailand	LC805701	KF758523	99.7	Clade II	IIa	This study
153	*P. insidiosum*	Pi013C2	Human	Thailand	LC805698		99.7	Clade II	IIa	This study
154	*P. insidiosum*	Pi012C2	Human	Thailand	LC805697		99.7	Clade II	IIa	This study
155	*P. insidiosum*	SIMI 620070693	Human	Thailand	KF758512		99.7	Clade II	IIa	GenBank
156	*P. insidiosum*	MAPI-1	Dog	Mali	OM334795		99.2	Clade II	IIa	[8]
157	*P. insidiosum*	PCM2	Human	Thailand	GQ451591		99.0	Clade II	IIa	[14]
158	*P. insidiosum*	MTP-75	Human	India	OM334799		99.5	Clade II	IIb	[8]
159	*P. insidiosum*	MTP-74	Human	India	OM334796		99.5	Clade II	IIb	[8]
160	*P. insidiosum*	MTP-78	Human	India	OM334803		99.5	Clade II	IIb	[8]
161	*P. insidiosum*	Pi038C2	Human	India	LC805716	OP160029	99.5	Clade II	IIb	This study
162	*P. insidiosum*	Pi40	Mosquito larva	India	LC553030		99.5	Clade II	IIb	[4]
163	*P. insidiosum*	RCB04	Environment	Thailand	LC549518		99.5	Clade II	IIb	[4]
164	*P. insidiosum*	KCB10	Environment	Thailand	LC549515		99.5	Clade II	IIb	[4]
165	*P. insidiosum*	KCB07	Environment	Thailand	LC549512		99.5	Clade II	IIb	[4]
166	*P. insidiosum*	KCB05	Environment	Thailand	LC549510		99.5	Clade II	IIb	[4]
167	*P. insidiosum*	KCB02	Environment	Thailand	LC549507		99.5	Clade II	IIb	[4]
168	*P. insidiosum*	SIMI 6666	Human	Thailand	KF758535		99.5	Clade II	IIb	GenBank
169	*P. insidiosum*	Pi23	Human	Thailand	LC553038	KF758521	99.5	Clade II	IIb	[4]
170	*P. insidiosum*	Pi026C2	Human	Thailand	LC805709	KF758504	99.5	Clade II	IIb	This study
171	*P. insidiosum*	Pi027C2	Human	Thailand	LC805710	KF758501	99.5	Clade II	IIb	This study
172	*P. insidiosum*	Pi028C2	Human	Thailand	LC805711		99.5	Clade II	IIb	This study
173	*P. insidiosum*	Pi107C2	Human	Thailand	LC805757		99.5	Clade II	IIb	This study
174	*P. insidiosum*	Pi095C2	NA	Thailand	LC805745		99.5	Clade II	IIb	This study
175	*P. insidiosum*	Pi096C2	NA	Thailand	LC805746		99.5	Clade II	IIb	This study
176	*P. insidiosum*	MTP-79	Human	India	OM334802		99.2	Clade II	IIb	[8]
177	*P. insidiosum*	MTP-76	Human	India	OM334801		99.2	Clade II	IIb	[8]
178	*P. insidiosum*	MTP-98	Human	India	OM334800		99.2	Clade II	IIb	[8]
179	*P. insidiosum*	MTP-81	Human	India	OM334797		99.2	Clade II	IIb	[8]
180	*P. insidiosum*	MTP-77	Human	India	OM334798		99.2	Clade II	IIb	[8]
181	*P. insidiosum*	PECM7	Environment	Thailand	GQ451580		99.2	Clade II	IIb	[14]
182	*P. insidiosum*	PMR3	Human	Thailand	GQ451584		99.2	Clade II	IIb	[14]
183	*P. insidiosum*	PECM11	Environment	Thailand	GQ451582		99.0	Clade II	IIb	[14]
184	*P. insidiosum*	PECM12	Environment	Thailand	GQ451596		99.0	Clade II	IIb	[14]
185	*P. insidiosum*	PECM15	Environment	Thailand	GQ451598		98.4	Clade II	IIb	[14]
186	*P. insidiosum*	PECM22	Environment	Thailand	GQ451592		98.4	Clade II	IIb	[14]
187	*P. insidiosum*	PECM9	Environment	Thailand	GQ451602		98.2	Clade II	IIb	[14]
188	*P. insidiosum*	PECM8	Environment	Thailand	GQ451581		97.9	Clade II	IIb	[14]
189	*P. insidiosum*	PECM14	Environment	Thailand	GQ451597		97.9	Clade II	IIb	[14]
190	*P. insidiosum*	PECM10	Environment	Thailand	GQ451603		97.4	Clade II	IIb	[14]
191	*P. insidiosum*	PECM17	Environment	Thailand	GQ451600		97.4	Clade II	IIb	[14]
192	*P. insidiosum*	AEH_6844	Human	India	MK424855		96.0	Clade II	IIb	GenBank
193	*P. insidiosum*	AEH_6232	Human	India	MK424854		95.7	Clade II	IIb	GenBank
194	*P. insidiosum*	PECM21	Environment	Thailand	GQ451593		95.5	Clade II	IIb	[14]
195	*P. insidiosum*	AEH_3453	Human	India	MK424851		94.4	Clade II	IIb	GenBank
196	*P. insidiosum*	Pi048C3	Human	Thailand	LC805721		Reference	Clade III	IIIa	This study
197	*P. periculosum*	MTP-80	Human	India	OM334805		100	Clade III	IIIa	[8]
198	*P. periculosum*	MTP-99	Human	India	OM334806		100	Clade III	IIIa	[8]
199	*P. periculosum*	MTP-72	Human	India	OM334807		100	Clade III	IIIa	[8]
200	*P. insidiosum*	KCB01	Environment	Thailand	LC549506		100	Clade III	IIIa	[4]
201	*P. insidiosum*	KCB03	Environment	Thailand	LC549508		100	Clade III	IIIa	[4]
202	*P. insidiosum*	KCB04	Environment	Thailand	LC549509		100	Clade III	IIIa	[4]
203	*P. insidiosum*	KCB06	Environment	Thailand	LC549511		100	Clade III	IIIa	[4]
204	*P. insidiosum*	KCB08	Environment	Thailand	LC549513		100	Clade III	IIIa	[4]
205	*P. insidiosum*	KCB09	Environment	Thailand	LC549514		100	Clade III	IIIa	[4]
206	*P. insidiosum*	BKDZ01	Environment	Thailand	LC549516		100	Clade III	IIIa	[4]
207	*P. insidiosum*	BKDZ02	Environment	Thailand	LC549517		100	Clade III	IIIa	[4]
208	*P. insidiosum*	PECM6	Environment	Thailand	GQ451579		100	Clade III	IIIa	[14]
209	*P. insidiosum*	Pi075C3	Human	Thailand	LC805740		100	Clade III	IIIa	This study
210	*P. insidiosum*	Pi057C3	Human	Thailand	LC805727		100	Clade III	IIIa	This study
211	*P. insidiosum*	Pi47	Human	Thailand	LC553044		100	Clade III	IIIa	[4]
212	*P. insidiosum*	MCC 6	Human	Thailand	KF758487		100	Clade III	IIIa	GenBank
213	*P. insidiosum*	SIMI 820070255	Human	Thailand	KF758513		100	Clade III	IIIa	GenBank
214	*P. insidiosum*	SIMI 1020142275	Human	Thailand	KF758515		100	Clade III	IIIa	GenBank
215	*P. insidiosum*	SIMI 620035970	Human	Thailand	KF758516		100	Clade III	IIIa	GenBank
216	*P. insidiosum*	SIMI 240-37	Human	Thailand	KF758526		100	Clade III	IIIa	GenBank
217	*P. insidiosum*	SIMI 1839-46	Human	Thailand	KF758528		100	Clade III	IIIa	GenBank
218	*P. insidiosum*	SIMI 322-37	Human	Thailand	KF758527		100	Clade III	IIIa	GenBank
219	*P. insidiosum*	SIMI 16068	Human	Thailand	KF758539		100	Clade III	IIIa	GenBank
220	*P. insidiosum*	Pi46	Human	Thailand	LC553045	KF758531	100	Clade III	IIIa	[4]
221	*P. insidiosum*	Pi105C3	Human	Thailand	LC805755		100	Clade III	IIIa	This study
222	*P. insidiosum*	PR2	Human	Thailand	GQ451574		100	Clade III	IIIa	[14]
223	*P. insidiosum*	Pi50	Human	USA	LC553027		100	Clade III	IIIa	[4]
224	*P. periculosum*	MTPI-35	Human	Israel	OM363735		99.7	Clade III	IIIa	[8]
225	*P. insidiosum*	MCC 1	Human	Thailand	KF758483		99.7	Clade III	IIIa	GenBank
226	*P. insidiosum*	PC7	Human	Thailand	GQ451588		99.7	Clade III	IIIa	[14]
227	*P. insidiosum*	A1SP	Environment	Brazil	MT939910		99.2	Clade III	IIIb	GenBank
228	*P. insidiosum*	CBS119454	Human	Brazil	MK000402		99.2	Clade III	IIIb	GenBank
229	*P. insidiosum*	MTPI19	Horse	Costa Rica	GQ451570		99.2	Clade III	IIIb	[14]

230	*P. insidiosum*	Pi051C3	Environment	Thailand	LC805722	OP168826	99.2	Clade III	IIIb	This study
231	*P. insidiosum*	Pi101C3	Environment	Thailand	LC805751		99.2	Clade III	IIIb	This study
232	*P. insidiosum*	PC1	Human	Thailand	GU479367		99.2	Clade III	IIIb	GenBank
233	*P. insidiosum*	PC3	Human	Thailand	GQ451573		99.2	Clade III	IIIb	[14]
234	*P. insidiosum*	SIMI 6655-48	Human	Thailand	KF758534		99.2	Clade III	IIIb	GenBank
235	*P. insidiosum*	MCC 14	Human	Thailand	KF758492		99.2	Clade III	IIIb	GenBank
236	*P. insidiosum*	SIMI 5141-48	Human	Thailand	KF758533		99.2	Clade III	IIIb	GenBank
237	*P. insidiosum*	Pi49	Human	Thailand	LC553046	KF758536	99.2	Clade III	IIIb	[4]
238	*P. insidiosum*	Pi44	Human	Thailand	LC553037		99.2	Clade III	IIIb	[4]
239	*P. insidiosum*	Pi45	Human	Thailand	LC553036	KF758491	99.2	Clade III	IIIb	[4]
240	*P. insidiosum*	Pi094C3	Human	Thailand	LC805744		99.2	Clade III	IIIb	This study
241	*P. insidiosum*	AEH_9479	Human	India	MK424860		98.4	Clade III	IIIb	GenBank
242	*P. insidiosum*	PECM20	Environment	Thailand	GQ451595		98.2	Clade III	IIIb	[14]
243	*P. insidiosum*	PECM13	Environment	Thailand	GQ451594		97.6	Clade III	IIIb	[14]
244	*P. periculosum*	MTP-82	Human	India	OM334804		97.4	Clade III	IIIb	[8]
245	*P. insidiosum*	AEH_8818	Human	India	MK424859		97.2	Clade III	IIIb	GenBank
246	*P. insidiosum*	AEH_8186	Human	India	MK424857		95.3	Clade III	IIIb	GenBank
247	*P. insidiosum*	AEH_52692	Human	India	MK424852		95.3	Clade III	IIIb	GenBank
248	*P. periculosum*	ATCC 90478	Bear	USA	OM363736		Reference	Clade IV	-	[8]
249	*P. periculosum*	MTPI-46	Dog	USA	OK493461		100.0	Clade IV	-	GenBank
250	*P. catenulatum*	RM9_06	Environment	Thailand	LC553642		86.8-92	Outgroup	-	[4]
251	*P. rhizo-oryzae*	RCB01	Environment	Thailand	LC553641		85.3-91	Outgroup	-	[4]

References

4. Mar Htun, Z.; Laikul, A.; Pathomsakulwong, W.; Yurayart, C.; Lohnoo, T.; Yingyong, W.; Kumsang, Y.; Payattikul, P.; Sae-Chew, P.; Rujirawat, T.; et al. Identification and Biotyping of *Pythium insidiosum* Isolated from Urban and Rural Areas of Thailand by Multiplex PCR, DNA Barcode, and Proteomic Analyses. *J. Fungi* **2021**, 7, 242. doi:10.3390/jof7040242.
8. Miraglia, B.M.; Mendoza, L.; Rammohan, R.; Vilela, L.; Vilela, C.; Vilela, G.; Huebner, M.; Mani, R.; Vilela, R. *Pythium insidiosum* Complex Hides a Cryptic Novel Species: *Pythium periculosum*. *Fungal. Biol.* **2022**, 126, 366–374. doi:10.1016/j.funbio.2022.03.002.
14. Kammarnjesadakul, P.; Palaga, T.; Sritunyalucksana, K.; Mendoza, L.; Krajaejun, T.; Vanittanakom, N.; Tongchusak, S.; Denduangboripant, J.; Chindamporn, A. Phylogenetic Analysis of *Pythium insidiosum* Thai Strains Using Cytochrome Oxidase II (COX II) DNA Coding Sequences and Internal Transcribed Spacer Regions (ITS). *Med. Mycol.* **2011**, 49, 289–295. doi:10.3109/13693786.2010.511282.
